# Erythema Nodosum Leprosum Triggered by Herpes Simplex Infection: A Case Report

**DOI:** 10.7759/cureus.98854

**Published:** 2025-12-09

**Authors:** Usamah Al-Anbagi, Abdulqadir J Nashwan, Samir Al Hyassat, Farah J Assaf, Hatem A Abdulmajeed, Abdelkarim H Mohamed

**Affiliations:** 1 Department of Internal Medicine, Hamad Medical Corporation, Doha, QAT; 2 Department of Nursing and Midwifery Research, Hamad Medical Corporation, Doha, QAT; 3 Department of Laboratory Medicine and Pathology, Hamad Medical Corporation, Doha, QAT; 4 Department of Clinical Imaging, Hamad Medical Corporation, Doha, QAT

**Keywords:** case report, erythema nodosum leprosum (enl), herpes simplex virus, lepromatous leprosy, mycobacterium leprae, type 2 lepra reaction

## Abstract

Leprosy, caused by *Mycobacterium leprae*, is a chronic granulomatous disease primarily affecting the skin and peripheral nerves. Erythema nodosum leprosum (ENL), or type 2 lepra reaction, is an immunologic complication of lepromatous leprosy, often presenting with erythematous nodules, fever, and systemic inflammation. Early recognition is crucial to prevent chronic complications. We report a 44-year-old man who developed facial erythematous nodules and fever following a sore throat, initially suggestive of a drug eruption or viral infection. Laboratory tests revealed neutrophilia, elevated inflammatory markers, mild transaminitis, and positive herpes simplex virus IgM. Imaging showed right periorbital soft tissue thickening. Skin biopsy confirmed lepromatous leprosy, and the rash was identified as ENL, likely triggered by acute herpes simplex virus infection. The patient received antibiotics followed by anti-leprosy therapy (dapsone, clofazimine, rifampicin) and tapering corticosteroids, with complete resolution of symptoms. This case highlights the importance of considering leprosy and its immunologic reactions in acute facial eruptions.

## Introduction

Leprosy, also known as Hansen’s disease, is a chronic granulomatous infection caused by *Mycobacterium leprae* and, less frequently, *Mycobacterium lepromatosis* [1]. The disease primarily targets the skin and peripheral nerves, leading to sensory impairment, neuropathy, and progressive deformities if left untreated. Despite being classified as a neglected tropical disease, leprosy remains a major health concern, with nearly 200,000 new cases reported annually worldwide [2,3]. India, Brazil, and Indonesia account for the majority of these cases, highlighting their ongoing burden in endemic regions [4]. Transmission is thought to occur mainly through respiratory droplets, although zoonotic transmission via armadillos has been documented in some regions [5].

An important clinical aspect of leprosy is the occurrence of acute inflammatory episodes termed lepra reactions, which significantly contribute to morbidity. The following two major types are recognized: type I (reversal reaction), which occurs due to changes in cell-mediated immunity, and type II, also known as erythema nodosum leprosum (ENL) [6]. ENL is an immune-complex-mediated hypersensitivity reaction seen in multibacillary forms of leprosy, particularly in lepromatous and borderline lepromatous disease. Clinically, ENL is characterized by crops of tender, erythematous nodules that may be accompanied by systemic features, such as fever, malaise, arthritis, lymphadenitis, orchitis, or nephritis [6].

ENL is not only common but clinically significant because of its recurrent and sometimes chronic course, which can lead to cumulative nerve damage and long-term disability [6]. Diagnosis can be challenging, as ENL may mimic more common dermatologic and infectious conditions, including viral exanthems, drug eruptions, neutrophilic dermatoses, or bacterial cellulitis [7]. Early recognition and prompt management are essential to prevent complications.

We report a case of lepromatous leprosy presenting with erythema nodosum leprosum, triggered by acute herpes simplex virus infection and complicated by secondary facial cellulitis. This case underscores the importance of maintaining a high index of suspicion for leprosy-related reactions and highlights the value of multidisciplinary management.

## Case presentation

History

A 44-year-old man with no chronic illnesses presented with a five-day history of sore throat and fever. He denies a history of cough, rhinorrhea, or shortness of breath. Three days later, he developed a facial rash, initially appearing on the left forehead, spreading to the right forehead, and subsequently involving the periorbital and maxillary regions. The lesions were raised, erythematous, painless, and non-pruritic, with the genital area and limbs spared. He denied mucosal involvement, visual disturbances, headache, back pain, joint pain, numbness, tingling, abdominal pain, diarrhea, or weight loss. He reported no prior history of similar eruptions, psoriasis, autoimmune disease, or leprosy, and no history of chronic skin lesions or limb numbness. There was no recent travel or sick contacts. He had no new medications or herbal use and no known allergies.

Examination

**Figure 1:**
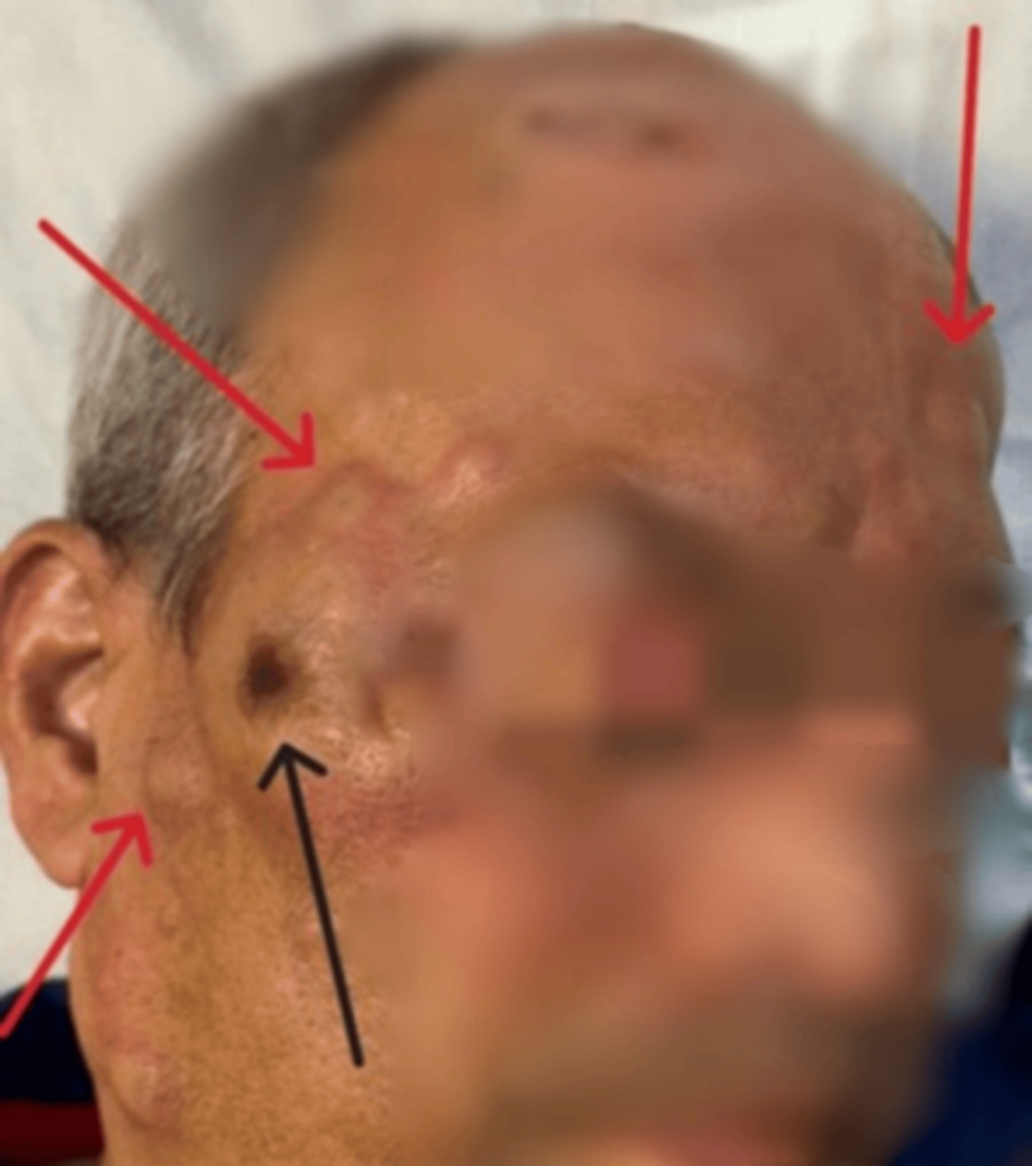
Erythema nodosum leprosum lesions with multiple nodules (red arrows); one nodule shows central necrosis (black arrow).

On presentation, the patient was alert and comfortable, not in distress. Vital signs were temperature 37°C, heart rate 89 beats per minute, respiratory rate 17 breaths per minute, blood pressure 117/60 mmHg, and SpO₂ 97% on room air. Dermatologic examination showed scattered erythematous papules and nodules of varying sizes, some with central necrosis, predominantly involving the face, including the forehead and cheeks. A notable lesion was present on the right cheek below the eye, with central necrosis and surrounding swelling, but without orbital pain (Figure 1).

Similar, though less prominent, lesions were scattered on the arms, chest, and legs, with the genital area spared. All lesions were painless and non-pruritic. Chest auscultation was clear bilaterally with no crackles, and cardiovascular examination revealed normal S1 and S2 and no murmurs. Abdominal examination was soft, non-tender, and no organomegaly; neurological examination was unremarkable, with no thickened or palpable peripheral nerves.

Management and hospital course

**Figure 2:**
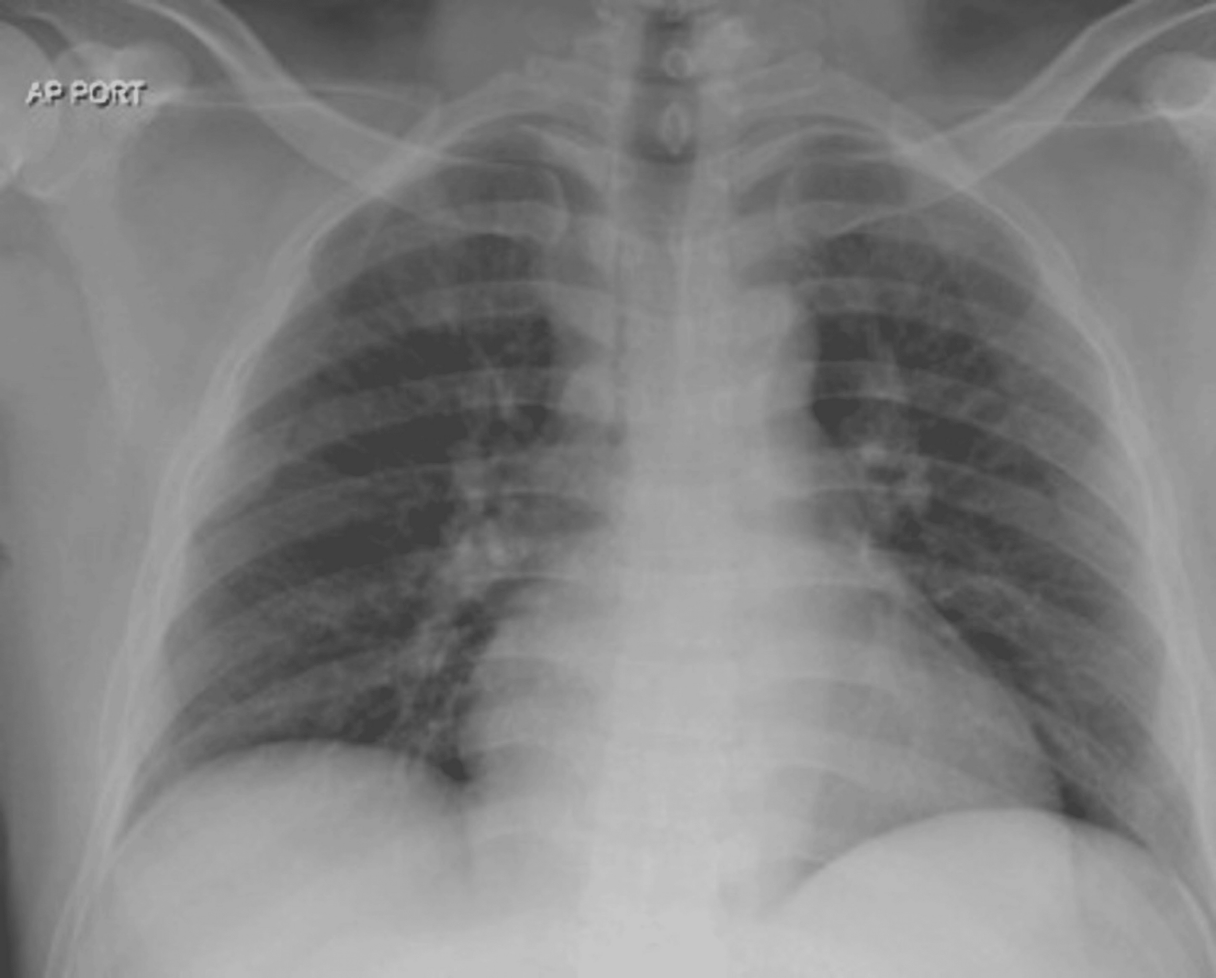
Chest X-ray, AP view, revealed an unremarkable study.

**Figure 3:**
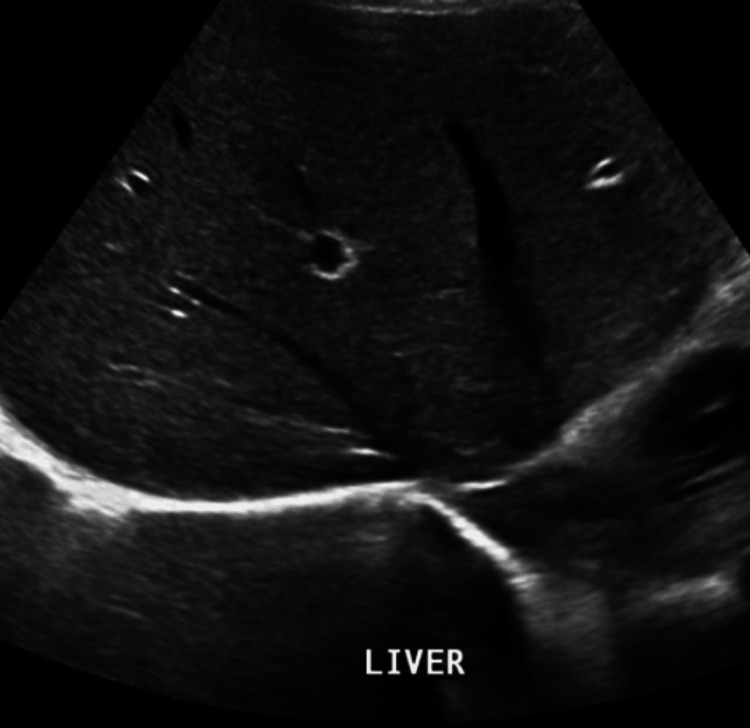
Abdominal ultrasound revealed an unremarkable study except for fatty liver changes.

Initial laboratory investigations revealed leukocytosis with neutrophilia, mild anemia, transaminitis, and elevated CRP, while initial viral antigen tests were negative (Table 1). The patient was admitted as a possible case of neutrophilic dermatosis, monkeypox (MPOX), drug eruption, or ENL, and placed in an isolation room. Full viral serologies were sent. Empirical antibiotic therapy with ceftriaxone 2 g daily was initiated. Initial radiological evaluation, including chest X-ray and abdominal ultrasound, was unremarkable except for fatty liver changes (Figures 2, 3).

**Table 1:** Summary of the patient’s laboratory results on admission and at discharge. CBC: complete blood count; ALT: alanine aminotransferase; AST: aspartate aminotransferase

Parameters	On admission	On discharge	Reference values
Total leukocytes (x10^3^/μL)	20	4.3	4-11×10³
Hemoglobin (g/dL)	12.7	13	13-17
Platelet (x10^3^/μL)	385	792	150-410
CRP (mg/L)	337	4.4	0-5
Procalcitonin (ng/mL)	0.23	Not done	<0.05
Serum urea (mmol/L)	3.6	5.3	2.5-7.8
Serum creatinine (μmol/L)	71	60	62-106
Serum sodium (mmol/L)	132	136	133-146
Serum potassium (mmol/L)	3.4	4.3	3.5-5.3
Serum calcium (mmol/L)	2.51	2.6	2.2-2.6
Serum total protein (g/L)	75	79	60-80
Serum albumin (g/L)	28	31	35-50
Alkaline phosphatase (U/L)	455	186	40-129
ALT (IU/L)	105	56	0-41
AST (IU/L)	62	30	0-41
Serum total bilirubin (mg/dL)	19	7	0-21

**Figure 4:**
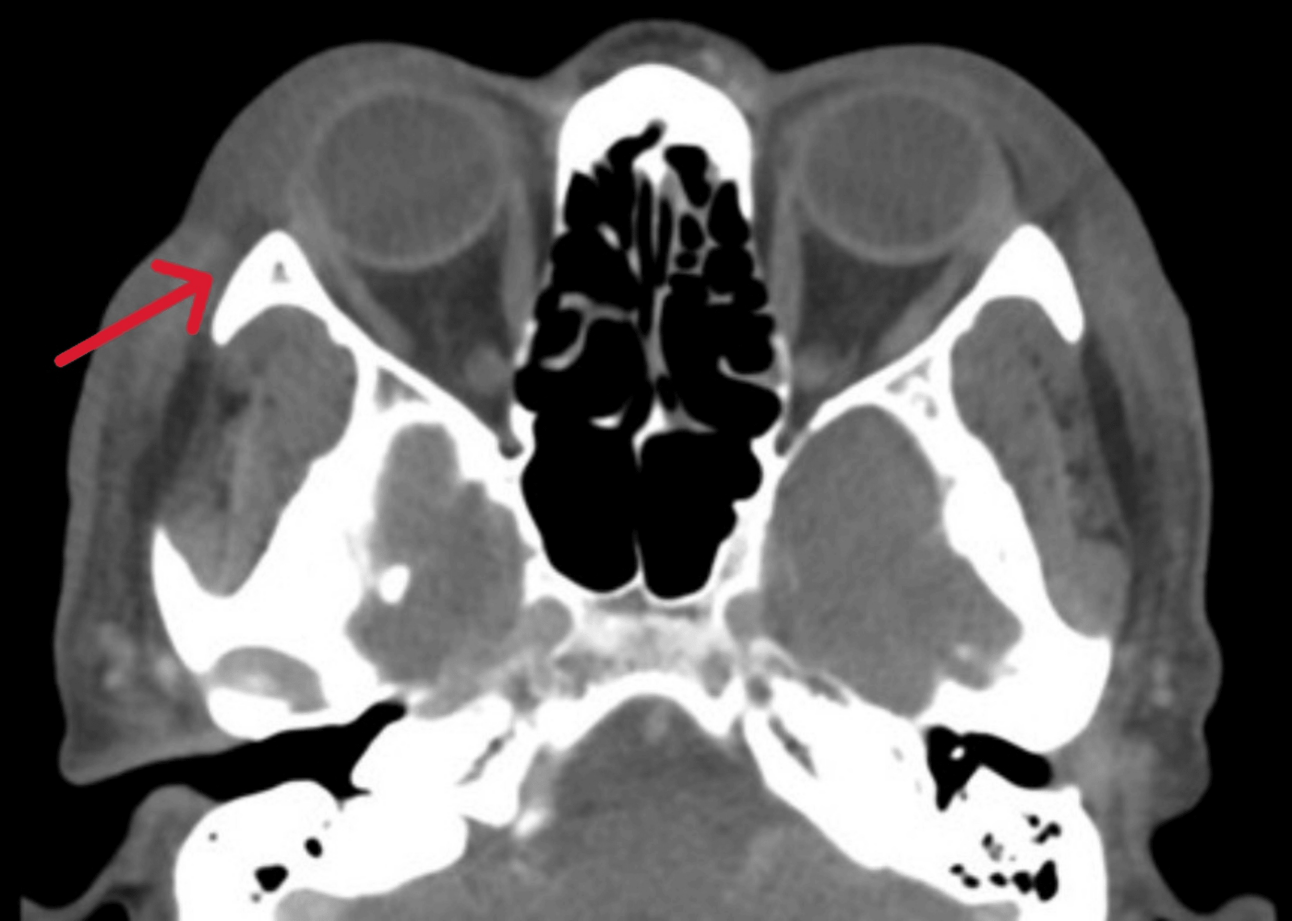
CT scan axial view revealed right periorbital (pre-septal) soft tissue thickening (red arrow).

The following day, viral serology showed positive IgM for herpes simplex virus, indicating an acute infection. Based on infectious disease team advice, antibiotics were escalated to piperacillin-tazobactam 4.5 g every 8 h, along with vancomycin 1 g every 8 h. The dermatology team recommended a biopsy of the lesion to confirm the diagnosis. Subsequent imaging included CT of the face, which demonstrated right periorbital (pre-septal) soft tissue thickening without abscess or infraorbital extension, and multiple bilateral subcentimeter cervical and left parotid enhancing lymph nodes (Figure 4).

**Figure 5:**
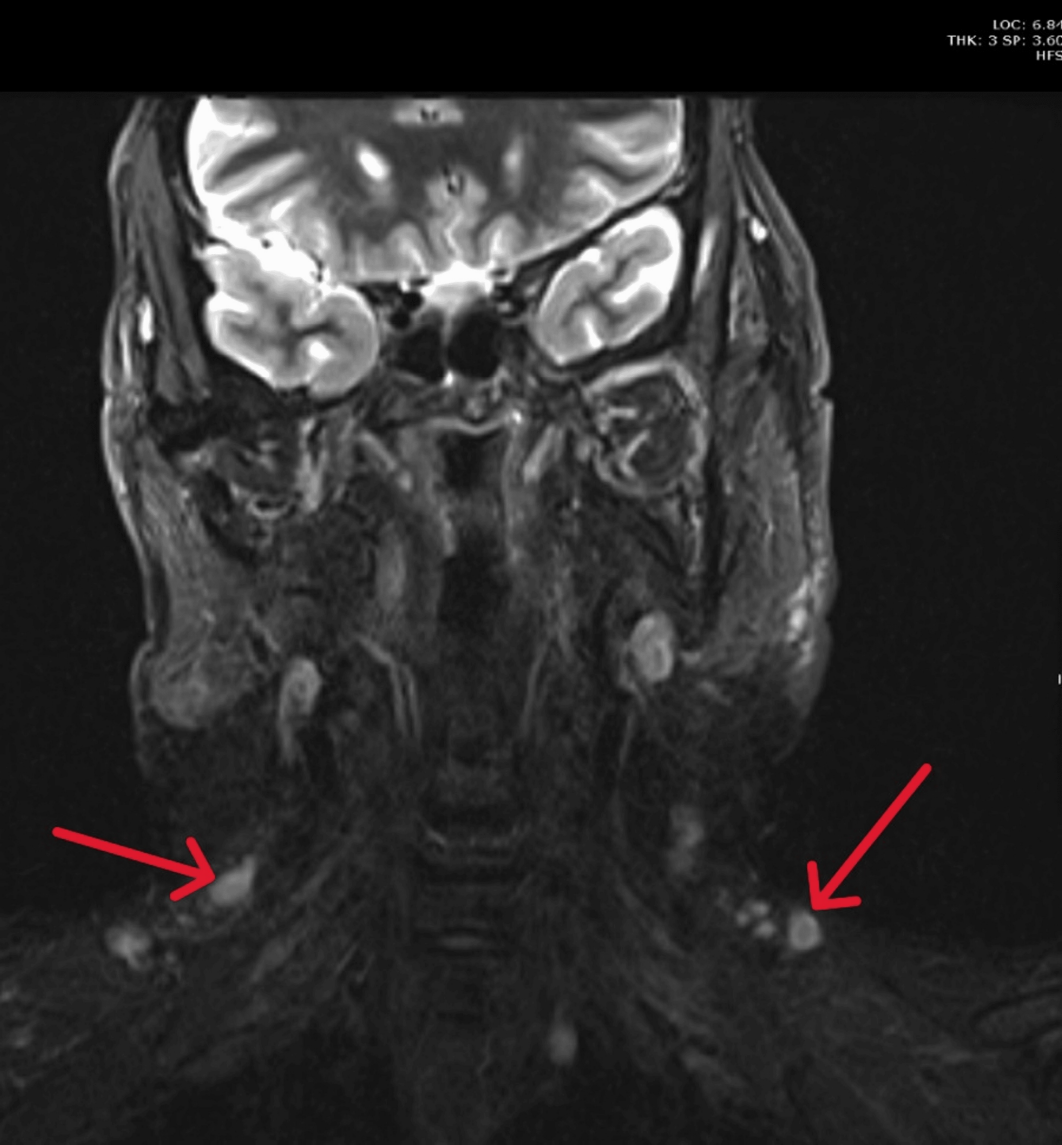
MRI of the neck revealed multilevel bilateral prominent lymph nodes, seen mainly within the supraclavicular regions bilaterally (red arrows).

**Figure 6:**
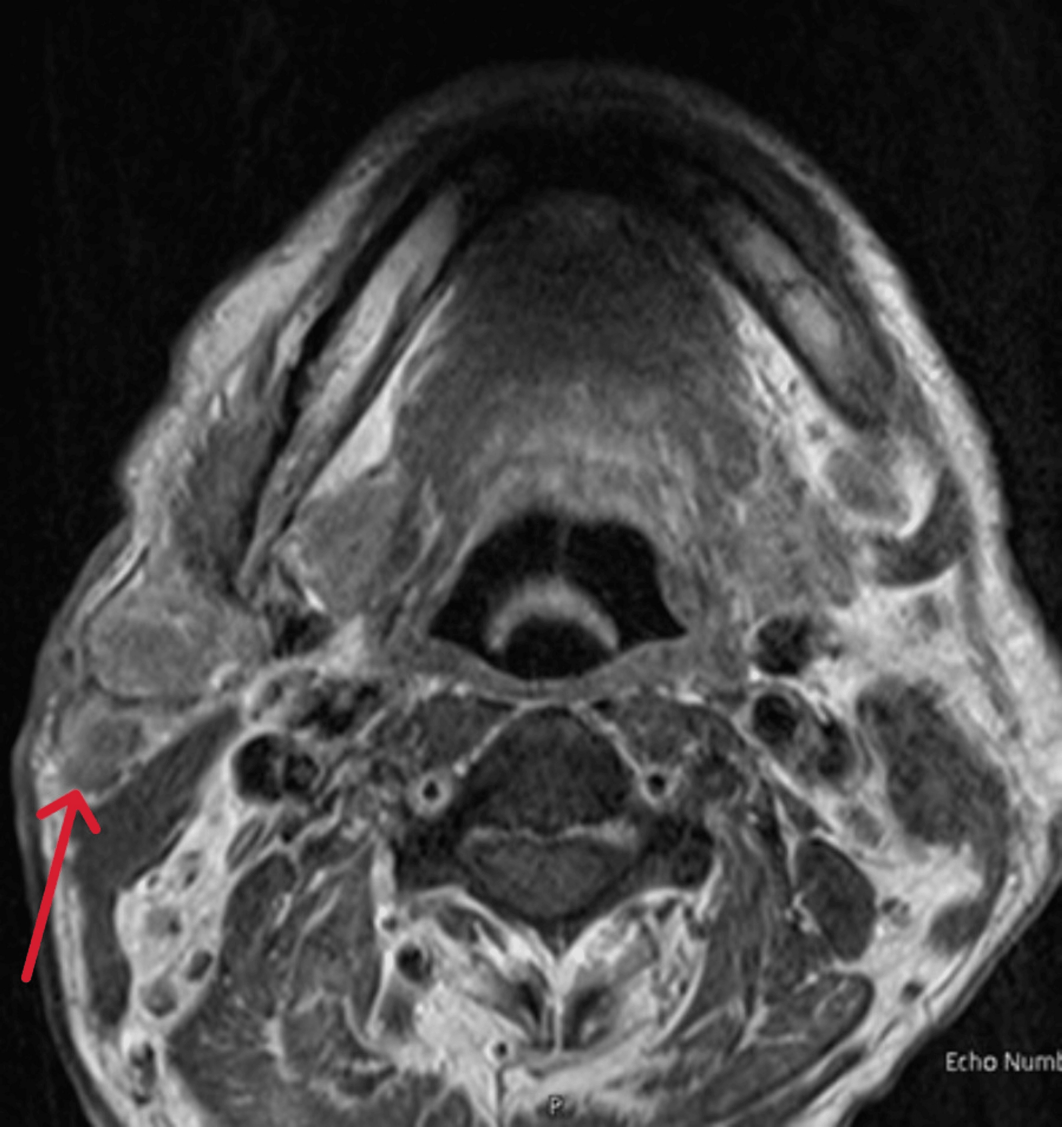
MRI of the face revealed central cystic necrosis right parotid gland (red arrow).

MRI of the neck and salivary glands revealed multilevel bilateral prominent lymph nodes (Figure 5), primarily in the parotid, submandibular, supraclavicular, and left jugulodigastric regions, with central cystic necrosis in some nodes (Figure 6).

**Figure 7:**
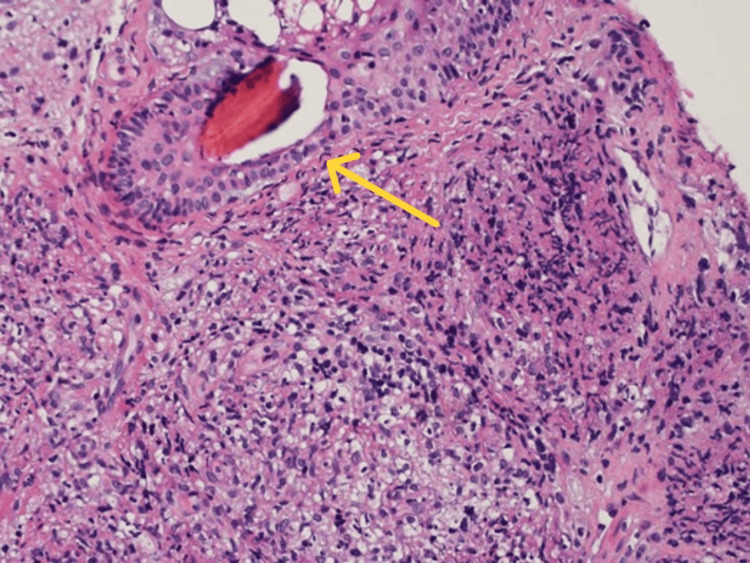
Hematoxylin and eosin stain showed dermal non-necrotizing granulomas with periadnexal and perineural distribution (200x) (yellow arrow).

**Figure 8:**
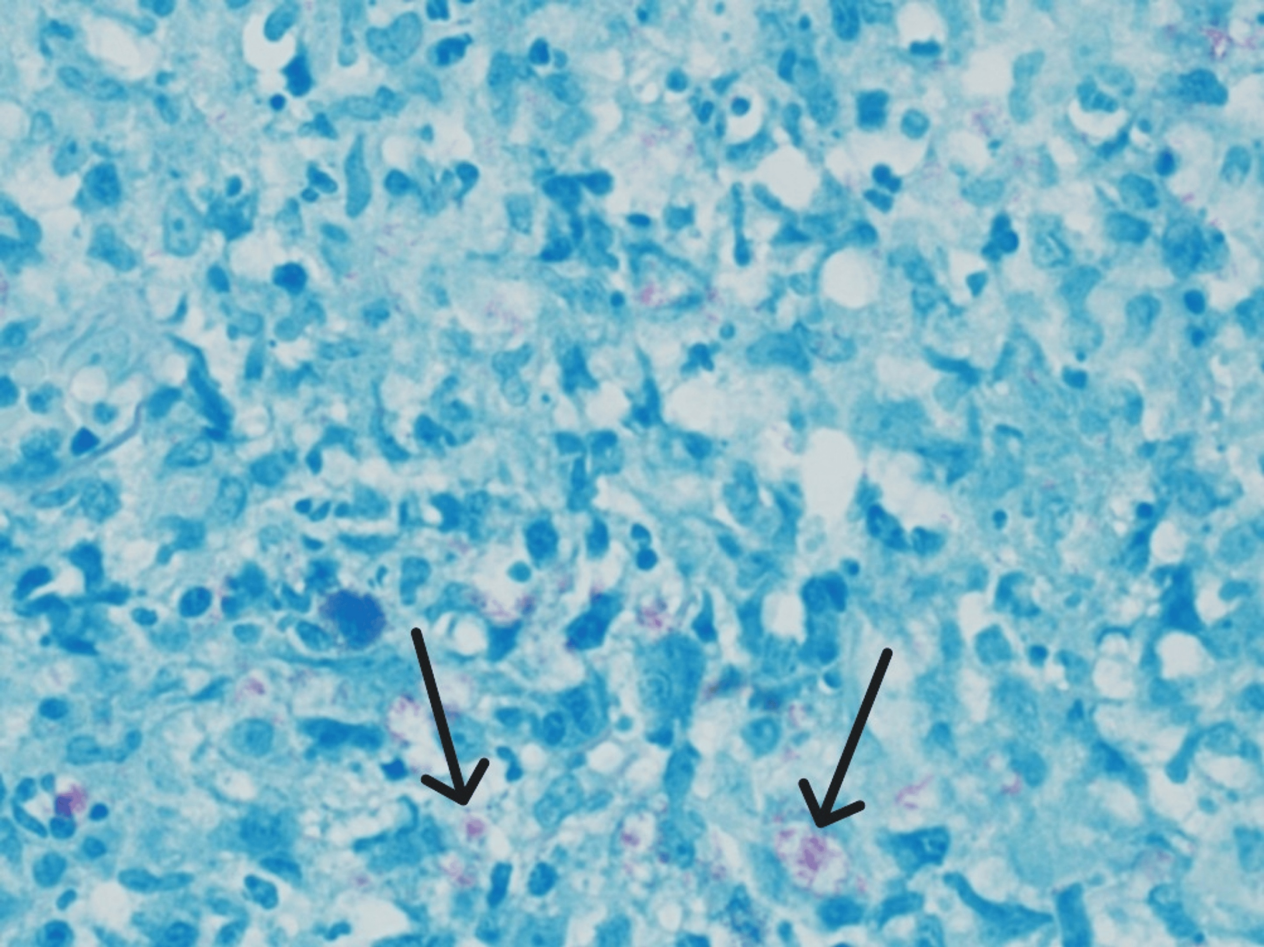
Wade-Fite special stain revealed numerous acid-fast bacilli within histiocytes (600x) (black arrows).

An excisional biopsy of the facial nodule was performed. Microscopically, the punch biopsy showed dermal non-necrotizing granulomas with periadnexal and perineural distribution. Wade-Fite and Ziehl-Neelsen special stains are positive for leprosy, lepromatous type (Figures 7, 8).

The final diagnosis was lepromatous leprosy with erythema nodosum leprosum, likely triggered by acute herpes simplex virus infection and complicated by secondary facial cellulitis. Herpes simplex virus IgM serology was positive, alternative ENL triggers were excluded, and the temporal overlap between acute HSV infection and ENL onset supports a causal association (Table 2) [8].

**Table 2:** Serological test results for viral infections. HSV: herpes simplex virus

Tests	Results	Interpretation
Herpes simplex virus IgM	Positive	Indicates recent or active HSV infection
Measles IgM	Negative	No evidence of recent measles infection
Varicella zoster IgM	Negative	No evidence of recent varicella zoster infection
Rubella IgM	Negative	No evidence of recent rubella infection
Parvovirus IgM	Negative	No evidence of recent parvovirus B19 infection
Mumps IgM	Negative	No evidence of recent mumps infection
Human immunodeficiency virus	Negative	No evidence of HIV infection
Hepatitis C virus antibody	Negative	No evidence of hepatitis C infection
Hepatitis B virus surface antigen	Negative	No evidence of active hepatitis B infection

The patient completed a 10-day course of intravenous antibiotics. Anti-leprosy therapy was delayed until discharge due to deranged liver function tests. At discharge, inflammatory markers had normalized, AST was normal, and ALT was near normal. He was prescribed dapsone 100 mg daily, clofazimine 50 mg daily, rifampicin 600 mg monthly, clofazimine 300 mg monthly, along with prednisolone 60 mg daily on a tapering protocol. At the six-day follow-up, the patient reported significant symptom resolution and no further lepra reactions. Regular follow-up has been scheduled to monitor treatment response and potential complications.

## Discussion

Leprosy continues to present significant diagnostic and management challenges despite the availability of effective multidrug therapy. Among its complications, ENL remains one of the most clinically significant due to its recurrent, multisystem nature and its potential to cause permanent nerve damage and disability [9]. ENL occurs in approximately 30-50% of patients with lepromatous or borderline lepromatous leprosy, representing an immune-complex-mediated type II hypersensitivity reaction [6,10-11]. The pathogenesis involves deposition of immune complexes, complement activation, and release of proinflammatory cytokines, such as tumor necrosis factor alpha (TNF-α), IL-6, and IL-1β, which drive both cutaneous and systemic inflammation [6].

In our patient, the presentation was unusual, as the lesions were painless, localized mainly to the face and periorbital region, and showed necrotic changes and cellulitic features, suggesting bacterial infection, drug reaction, or neutrophilic dermatosis. This atypical distribution, combined with the absence of classic nerve involvement at presentation, contributed to the diagnostic challenge. ENL typically manifests as crops of painful erythematous nodules on the extremities, trunk, and face, but can also mimic pustular or necrotizing dermatoses, as seen here [6,12].

The diagnostic process was further complicated by concurrent findings. The patient tested positive for herpes simplex virus (HSV) IgM, suggesting an acute viral infection. Viral triggers are recognized precipitants of lepra reactions, as immune activation can destabilize the delicate host-pathogen balance in multibacillary leprosy [6,12,13]. HSV, although rarely reported as a direct precipitant, may have contributed to the onset of ENL in this case [14]. Additionally, secondary bacterial cellulitis of the periorbital region obscured the underlying pathology, necessitating the use of empirical broad-spectrum antibiotics. Such overlapping conditions emphasize the need for careful, multidisciplinary assessment.

Management of ENL requires simultaneous treatment of the infection and the immunologic reaction. The cornerstone of leprosy treatment remains multidrug therapy (MDT) comprising rifampicin, dapsone, and clofazimine [6,10]. For acute ENL, systemic corticosteroids are the first-line therapy due to their rapid anti-inflammatory effect. Thalidomide is highly effective, particularly in recurrent or steroid-dependent ENL, but its teratogenicity restricts use, especially in women of childbearing age. Other steroid-sparing agents, such as methotrexate, azathioprine, or TNF-α inhibitors, have been explored in refractory cases. Clofazimine, a component of MDT, also has an anti-inflammatory effect and may help prevent recurrences with long-term use [10,15,16].

In our patient, initiation of MDT was delayed due to deranged liver function tests, necessitating stabilization before therapy. He was treated empirically with ceftriaxone followed by piperacillin-tazobactam and vancomycin for suspected cellulitis, while dermatology consultation led to biopsy and definitive diagnosis. Corticosteroid therapy was introduced to control ENL, with subsequent clinical improvement. This sequence highlights the importance of tailoring treatment to individual comorbidities while ensuring prompt initiation of anti-leprosy therapy once feasible.

The risk of recurrence remains significant, particularly in patients with high bacillary loads, delayed treatment, or intercurrent infections. Long-term follow-up is essential to monitor for relapse, neuropathy, and systemic complications [6,10]. This case underscores that ENL can present atypically, mimic other dermatologic or infectious conditions, and be triggered by external factors such as viral infections. Multidisciplinary collaboration is critical to achieve timely diagnosis and effective management.

## Conclusions

Erythema nodosum leprosum is a severe immunologic complication of lepromatous leprosy that can mimic other dermatologic and infectious conditions, leading to diagnostic delay. The present case highlights how viral infections, such as herpes simplex, may trigger ENL and complicate its presentation with secondary bacterial cellulitis. Early recognition, histopathological confirmation, and a multidisciplinary management strategy are essential to optimize outcomes and prevent long-term complications. This report reinforces the need for heightened awareness of lepra reactions, particularly in endemic settings, to facilitate prompt diagnosis and effective treatment.
